# Thin-film InGaAs metamorphic buffer for telecom C-band InAs quantum dots and optical resonators on GaAs platform

**DOI:** 10.1515/nanoph-2021-0552

**Published:** 2022-02-08

**Authors:** Robert Sittig, Cornelius Nawrath, Sascha Kolatschek, Stephanie Bauer, Richard Schaber, Jiasheng Huang, Ponraj Vijayan, Pascal Pruy, Simone Luca Portalupi, Michael Jetter, Peter Michler

**Affiliations:** Institut für Halbleiteroptik und Funktionelle Grenzflächen, Center for Integrated Quantum Science and Technology (IQST) and SCoPE, University of Stuttgart, Allmandring 3, 70569 Stuttgart, Germany

**Keywords:** 1550 nm, metamorphic buffer, optical resonators, quantum dots

## Abstract

The GaAs-based material system is well-known for hosting InAs quantum dots (QDs) with outstanding optical properties, typically emitting at a wavelength of around 900 nm. The insertion of a metamorphic buffer (MMB) can shift this emission to the technologically attractive telecom C-band range centered at 1550 nm. However, the thickness of common MMB designs (>1 μm) limits their compatibility with most photonic resonator types. Here, we report on the metal–organic vapor-phase epitaxy (MOVPE) growth of a novel InGaAs MMB with a nonlinear indium content grading profile designed to maximize plastic relaxation within minimal layer thickness. This allows us to achieve the necessary transition of the lattice constant and to provide a smooth surface for QD growth within 180 nm. Single-photon emission at 1550 nm from InAs QDs deposited on top of this thin-film MMB is demonstrated. The strength of the new design is proven by integrating it into a bullseye cavity via nano-structuring techniques. The presented advances in the epitaxial growth of QD/MMB structures form the basis for the fabrication of high-quality telecom nonclassical light sources as a key component of photonic quantum technologies.

## 1 Introduction

Semiconductor quantum dots (QDs) are an appealing choice for the role of nonclassical light source in quantum technologies. The combination of InAs QDs on GaAs substrates has proven to be especially promising [1]. The typical emission wavelength of this system lies around 900 nm due to the compressive strain causing a significant blue-shift, compared to the bulk emission of InAs. However, long-distance quantum information applications would highly profit from utilizing photons in the telecom C-band window (around 1550 nm), which allows low-loss transmission through the atmosphere [2] and the glass fiber network [3]. There are two main approaches to reduce the blue-shifting strain on InAs QDs and obtain emission at 1550 nm. The first is a change of the substrate from GaAs to InP [4, 5], and the second, the growth of a metamorphic buffer (MMB) below the QDs [6, 7]. Single-photon emission from InAs QDs grown on InP has been studied extensively for decades and significant advances have been reported in recent years [8, 9]. Nevertheless, from a device fabrication point of view, the MMB on GaAs approach offers distinct benefits: it avoids the introduction of phosphorous compounds into the structure and allows fine-tuning the strain via the lattice constant of the matrix, providing more freedom for the QD growth parameters. Additionally, it unlocks access to efficient binary AlAs/GaAs distributed Bragg reflectors (DBRs) as well as AlGaAs-based etch-stop and sacrificial layers for various processing techniques.

MMBs consisting of III-V materials are well-established in a wide range of semiconductor devices, such as high electron mobility transistors [10] and multi-junction solar cells [11]. The functionality of these devices is generally independent of the MMB thickness, provided that a high crystalline quality is ensured. Therefore, MMBs are typically at least 1 μm thick and their material composition is linearly or step-graded, because this approach facilitates control over properties like surface roughness and defect density [12].

Likewise, a linear InGaAs MMB with a thickness of 1080 nm enabled the first demonstration of single-photon emission in the telecom C-band from InAs QDs grown on the GaAs material platform [13]. Furthermore, polarization-entanglement [14], on-demand generation of entangled, single photon pairs [15] and indistinguishability under continuous-wave (cw) two-photon-resonant [16] and pulsed resonant [17] excitation was shown by utilizing the same design. However, this sample structure features only a nominal 3*λ*-cavity between bottom DBR and semiconductor/air interface. Employing an advanced photonic structure, e.g., a *λ*-cavity micro-pillar, would substantially improve the emission properties [18]. Therefore, enabling device fabrication is a crucial next step.

A suitable MMB design must fulfil three mandatory requirements. First, provide sufficient strain reduction to shift the QD emission to 1550 nm. Second, a smooth and homogeneous surface is a necessary prerequisite for all processing techniques. Third, in order to retain its compatibility with the AlAs/GaAs material system, the MMB must be placed inside the *λ*-cavity for most photonic structures. This puts a strict upper limit on its thickness. Consequently, the previous linear design is unsuitable for this purpose and a thin-film replacement has to promote an extremely efficient transition of the lattice constant, while maintaining a high crystalline quality.

## 2 Relaxation-optimized buffer design

**Figure 1:**
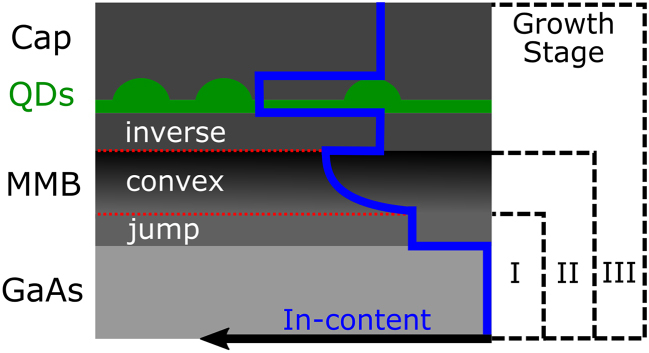
Schematic structure of InAs QDs inside an InGaAs-matrix lattice matched to an MMB grown on a GaAs substrate. The varying indium content throughout the structure is illustrated in blue. Growth optimization is performed in three consecutive growth stages I, II and III.

The intentional alteration of the lattice constant during metamorphic heteroepitaxy is achieved by inducing the formation of misfit dislocation segments into the previously pseudomorphic layer. Thus, the strained layer relaxes and adopts an in-plane lattice constant closer to its inherent value. Although the exact mechanisms responsible for the formation of these segments are only partly understood, this process is clearly driven by strain energy [19, 20]. Therefore, a large lattice mismatch with the substrate is desirable for the growth of a thin MMB. However, there is an upper limit for the possible mismatch, which is given by the onset of 3D-growth as a competing mechanism of strain energy reduction. Our proposed content grading profile to provide the maximum possible strain energy at any point, while staying within the limits of 2D-growth, is depicted by the InGaAs MMB in Figure 1. The grading begins with an abrupt change (jump) of the indium content, followed by a convex-up grading, and is completed by an inverse step. This jump-convex-inverse design is optimized in three successive growth stages named I, II and III.

In stage I, which corresponds to only the jump-layer, the aim is to induce a quick start of the plastic relaxation. Thus, we need to find the maximal permissible In content for this jump and the minimal thickness for the onset of relaxation.

Once the lattice constant begins to increase, the indium content can be increased further without generating 3D-structures. Following the same rationale, a convex-up function, in analogy to the metamorphic relaxation curve [21], is deduced as the steepest possible grading profile. This jump into convex-up design is compared with several alternatives in growth stage II. Additionally, the minimum thickness (i.e., maximum grading) that still maintains low surface roughness has to be determined. Furthermore, in order to reach the desired lattice constant, but avoid the stagnant saturation regime [22], an overshoot of In content is employed in the convex region before a decrease in the inverse region (see the dip at interface convex/inverse). Notably, this overshoot has to be adjusted retroactively to enable a lattice-matched deposition of the fully relaxed inverse layer.

The main role of the inverse layer is to serve as a substrate for the QD deposition and (together with the capping layer) to provide the correct amount of strain release to allow for the formation of InAs QDs with emission within the telecom C-band. This will therefore be the pivotal criterion for the optimization in stage III.

The key steps of the MMB growth calibration and optimization are presented in the following.

## 3 Optimization procedure

### 3.1 Jump layer optimization

**Figure 2:**
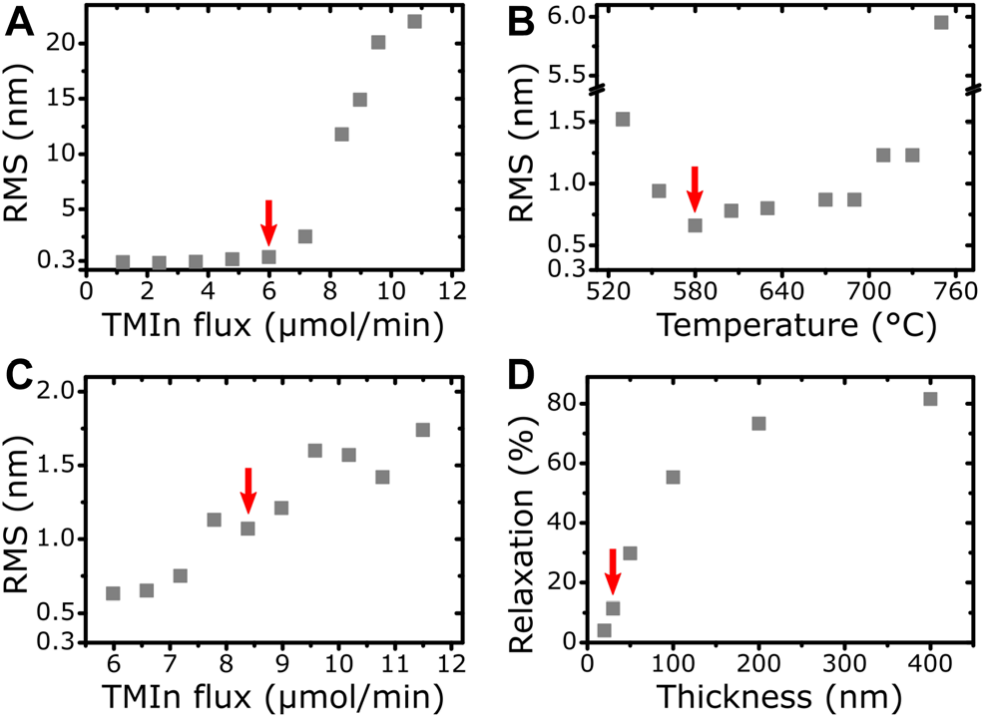
Successive optimization steps of growth stage I. The red arrows mark the obtained parameter values. (A–C) RMS surface roughness of InGaAs layers with varying growth parameters. (A) Variation of TMIn flux for the growth of 50 nm InGaAs with 20.8 μmol/min TMGa flux and 2973 μmol/min AsH_3_ flux at 710 °C. (B) Temperature dependence of InGaAs with a TMIn flux of 6.0 μmol/min. (C) Variation of TMIn flux at 580 °C. (D) Strain relaxation of In_0.274_GaAs depending on layer thickness.

We adopted the starting parameters for the optimization of the jump-layer (stage I) from our default metal–organic vapor-phase epitaxy (MOVPE) growth of high quality GaAs, namely a temperature of 710 °C, a TMGa flux of 20.8 μmol/min and an AsH_3_ flux of 2973 μmol/min. We then grew samples with 50 nm thick layers of InGaAs by adding varying amounts of TMIn into the mix. As shown in Figure 2A, the RMS surface roughness of these layers exhibits a sharp rise for TMIn fluxes greater than 7 μmol/min, indicating a transition towards 3D growth. In contrast, at 6 μmol/min we observe only a slight roughening compared to the baseline of 0.3 nm (GaAs grown in step-flow mode) while X-ray diffraction (XRD) analysis reveals metamorphic relaxation. We therefore selected this value for further investigation.

The next step was to examine the influence of the growth temperature, as this parameter is crucial for controlling the diffusion of atoms on the surface and the mobility of dislocations inside the layer [23]. A corresponding comparison of surface roughness is displayed in Figure 2B. Increasing the temperature from 710 °C to 750 °C leads to significant roughening. On the other hand, the RMS improves for lower temperature and only rises again below 550 °C, probably due to a general decline of epitaxial quality caused by insufficient pyrolysis of the precursors [24]. A growth temperature of 580 °C is found to be most advantageous, since it results in minimal surface roughness.

Next, we reiterated the determination of the maximum possible indium content, because a lower temperature is expected to suppress 3D-growth [25]. In contrast to the results at 710 °C, no clear transition can be identified, instead we observe an approximately linear relation between RMS and TMIn flux as shown in Figure 2C. However, the surface topography for higher indium contents, reveals ordering along the diagonal [001] and [010] directions (see Supplementary). In contrast, metamorphic layers typically exhibit a [011]/ $\left[0\bar{1}1\right]$ cross-hatch pattern [21]. Therefore, we decided to avoid this regime and adopted 8.4 μmol/min as the maximum applicable TMIn flux for the jump layer. This corresponds to an indium content of 27.4 ± 0.2% according to XRD analysis.

With the material composition and the temperature defined, the next step was to find the minimum thickness at which the layer starts to relax. For this purpose, we grew samples with InGaAs layer thicknesses ranging from 20 nm to 400 nm and determined their respective relaxation via XRD. The results are displayed in Figure 2D. The relaxation curve exhibits the typical behavior of metamorphic growth, namely a steep increase after a certain critical thickness, followed by saturation for thicker layers [22]. 30 nm is determined as the minimal thickness that exhibits a clear onset of relaxation (11.4%) and is therefore used as parameter for the jump-step layer. This completes the optimization of growth stage I.

### 3.2 Convex grading layer optimization

**Figure 3:**
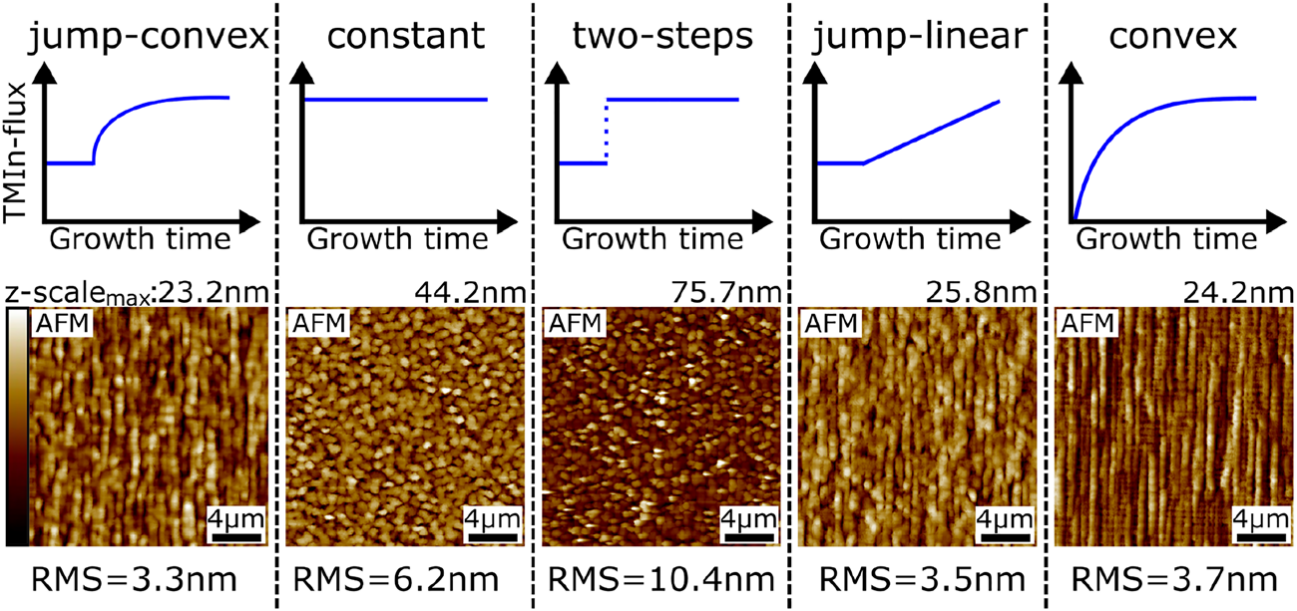
Optimization of growth stage II: Comparison between AFM-scans of various In_*x*_Ga_1−*x*_As grading profiles to reach 13.2 μmol/min TMIn flux. The constant section used in profiles 1, 3 and 4 has a thickness of 30 nm and the total thickness of each structure is 200 nm.

Figure 3 shows a comparison of our proposed jump-convex content-grading profile with various alternatives, each reaching a maximum TMIn flux of 13.2 μmol/min within 200 nm. Using a constant or two-steps profile is clearly inferior due to their higher surface roughness. Furthermore, employing a jump-linear or convex profile results in a similar RMS, but provides less average strain for the relaxation process. Therefore, we proceeded with finding a suitable thickness for the grading layer. Notably, a thinner convex segment steepens the necessary grading not only directly, but also indirectly by decreasing the final relaxation, which entails a larger overshoot to reach the desired effective lattice constant. 130 nm was determined as suitably thin, but still allowed adjusting the final indium content without inducing 3D-growth (see Supplementary). This is necessary for the next task of simultaneously fine-tuning the composition of convex and inverse/capping layer to obtain QD emission at 1550 nm in growth stage III.

### 3.3 Inverse layer optimization

**Figure 4:**
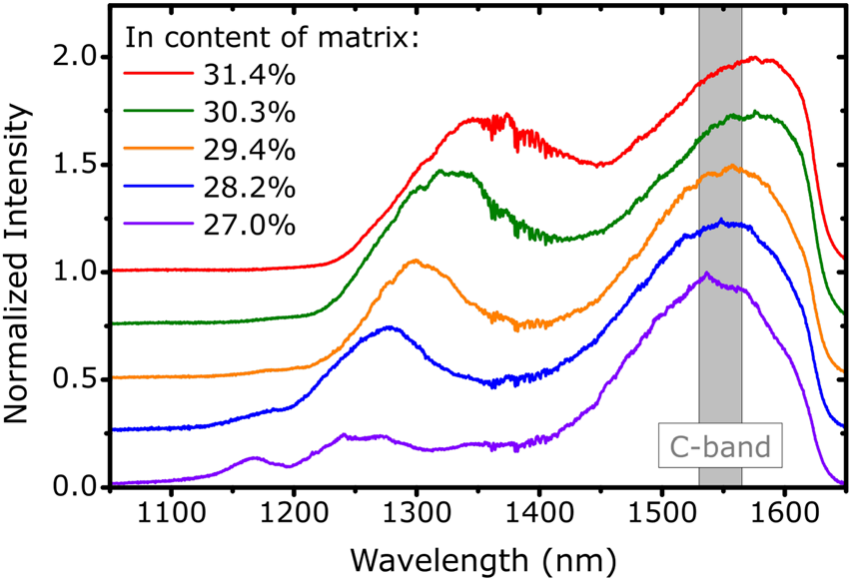
Optimization of growth stage III: Photoluminescence spectra of samples with varying indium content in the In_*x*_Ga_1−*x*_As matrix around the QDs. The curves are offset vertically for clarity.

For this purpose, we fabricated five capped samples with the indium content inside the matrix around the QDs ranging between 27.0% and 31.4% (35.9% to 40.6% at maximum of convex layer). The QDs were grown by depositing InAs for 4 s with a TMIn-flux of 9.6 μmol/min at 595 °C. The thickness of the inverse/capping layer was set to 60 nm/220 nm. This sets the QDs in the center of a structure with a total thickness of 440 nm, which corresponds to the approximate geometrical length of a *λ*-cavity consisting of the deposited InGaAs [26]. A comparison between the photoluminescence spectra of the five samples measured under nonresonant excitation at 4 K is shown in Figure 4. Each spectrum is composed of two peaks. The peak around 1550 nm can be identified as QD emission and the one at shorter wavelengths is assumed to stem from the wetting layer or the InGaAs matrix. As expected, the higher the In content of the matrix, the stronger the red-shift of the emission. However, the QD emission peak maximum varies by only 40 nm for the displayed composition range, compared to the inhomogeneously broadened ensemble distributions with a full width at half maximum (FWHM) of more than 150 nm. This means that the growth process is stable against small alterations of the indium content. Nevertheless, microphotoluminescence (*μ*-PL) measurements (not shown) revealed that the In_0.294_GaAs configuration produces the highest percentage of single QDs emitting in the 1545–1555 nm range. Hence, we selected this indium content as the most suitable, which finalized the optimization of the jump-convex-inverse design.

## 4 Photonic structures

In order to illustrate the potential and quality of the novel MMB we fabricated two exemplary structures.

**Figure 5:**
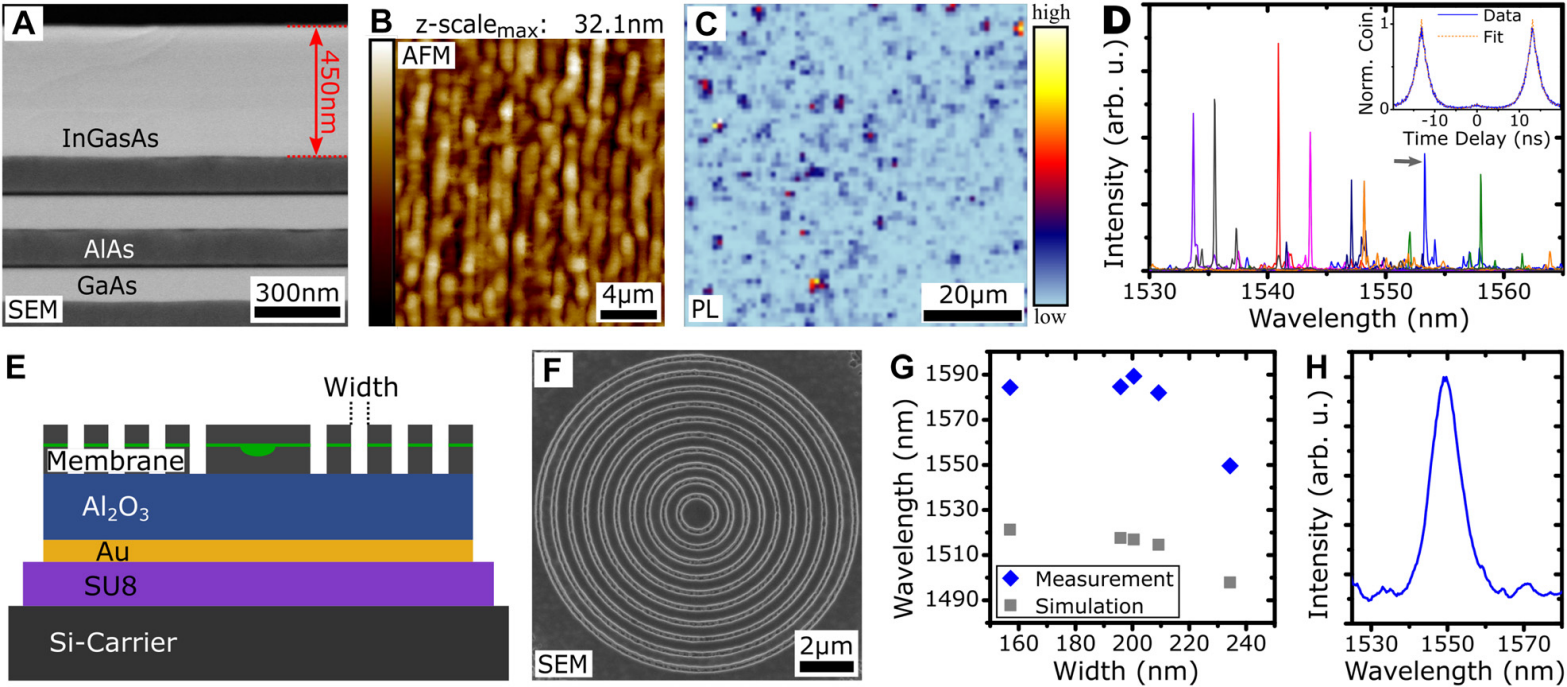
Exemplary QD/MMB structures. (A–D) Structural and optical investigation of a QD sample featuring an In_0.294_GaAs-matrix and an AlAs/GaAs DBR. (A) SEM side view of the two upper DBR-pairs, the jump-convex-inverse MMB and the capped QDs. (B) AFM scan showing the surface topography. The extracted RMS is 4.68 nm. (C) *μ*-PL map. The color scale represents the emission intensity inside the C-band. An area density for the QDs of 3 × [10^6^] cm^−2^ was extracted. (D) *μ*-PL spectra of eight exemplary QDs emitting inside the telecom C-band. The spectra are acquired under cw excitation apart from the blue spectrum (marked with a grey arrow) for which the second-order auto-correlation measurement under pulsed conditions (Inset) is acquired. (E–H) Bullseye structure for emission inside the telecom C-band. (E) Schematic of the sample after the flip-chip process. An Al_2_O_3_ spacer layer between the membrane and the backside gold mirror is used to enable constructive interference between the upwards and the backreflected emission. (F) SEM top view of an exemplary processed circular Bragg grating cavity. (G) Simulated and measured resonance wavelength of the fundamental cavity mode as a function of the trench width. (H) *μ*-PL spectrum of the cavity peak at 1550 nm under high power above band-gap pumping.

The first one was grown on top of 23 AlAs/GaAs DBR pairs for increased light extraction and also features QDs with adjusted growth parameters to account for the deposition on the jump-convex-inverse MMB. The SEM scan in Figure 5A displays a side view of the two upper DBR pairs, the MMB and the capped QDs. The InGaAs structure is 450 nm thick in total, which corresponds to the measured geometrical length for a *λ*-cavity as opposed to the approximated value used above. Notably, growth on AlAs instead of GaAs had no adverse influence on the quality of the MMB. The AFM scan in Figure 5B displays the surface topography of the sample. It is dominated by a cross-hatch pattern, which is typical for MMB structures [21], resulting in an RMS of 4.68 nm. This topography remains homogeneous over the whole sample. Figure 5C shows a *μ*-PL map of the sample under cw nonresonant excitation in which the color scale depicts the emission intensity inside the telecom C-band. An analysis of distinct emitters allows extracting an area density for optically active QDs of ∼3 × [10^6^] cm^−2^, which signifies excellent conditions for the excitation of isolated emitters, as required in quantum optical experiments. Furthermore, in contrast to the findings reported in Ref. [13], the QDs are distributed uniformly with no visible preferred formation in specific areas of the cross-hatch. This indicates the absence of sufficiently strong strain fields on top of the relaxed inverse layer to influence the island formation [27]. A selection of exemplary *μ*-PL spectra acquired under nonresonant excitation is shown in Figure 5D exhibiting multiple sharp transition lines which is typical for QDs under this excitation scheme. QD emission can be found over the full range of the C-band. Note that the measurements are taken under cw excitation except for the transition marked with the arrow (see figure caption). The intensity is comparable to the sample investigated in Ref. [13]. Since the decay time is comparable as well (see below), the brightness of the QDs in this sample structure can be estimated to ∼3% into an NA of 0.6 [16] (see Supplementary). The mean value of the linewidth of the brightest transitions of a set of 26 exemplary QDs under saturation power (in cw excitation) is found to be 65.8 μeV (15.9 GHz) (FWHM) with a standard deviation of 8.2 μeV (2.0 GHz). This is close to the resolution limit of the spectrometer of ∼ 41 μeV (10 GHz). A higher resolution is attainted by performing first-order correlation measurements on a set of 17 different, exemplary QDs under the same excitation conditions yielding a mean value of 46.7 μeV (11.3 GHz) (FWHM) with a standard deviation of 9.1 μeV (2.2 GHz) (see Supplementary). The lineshape is dominated by Gaussian broadening. Both width and shape of the transition lines compare well to QDs grown on a linearly graded MMB [16]. To gain insight into the fine-structure splitting (FSS) of the QDs, polarization-resolved *μ*-PL measurements are performed on 26 QDs. The mean value of the FSS is 23.4 μeV with a standard deviation of 20.6 μeV, thus a reasonable number of QDs exhibit an FSS below 10 μeV (see Supplementary). Note that this value is based in part on the polarization-dependent spectral shift of single transition lines, as a clear biexciton–exciton pair could only be identified in 6 out of 26 cases. The brightest transition line exhibits an FSS for 7 out of the 26 investigated QDs, suggesting the trion recombination to be favored, as was the case of the linearly graded MMBs [13, 28]. The QDs also compare well with respect to decay time [13, 16, 17]. Time-correlated single-photon counting measurements have been performed under weak, pulsed, nonresonant excitation for 13 exemplary QD transitions. The decay traces are predominantly mono-exponential with a mean decay time of 1.39 ns (standard deviation 0.16 ns). The nonclassical nature of the emitted light is demonstrated for an exemplary second-order auto-correlation function *g* ^(2)^(*τ*) under pulsed, nonresonant excitation and displayed in the inset of Figure 5D. The measurement was acquired with a mean, total count rate of 76.9 kcps, integrating for 45 min using superconducting nanowire single-photon detectors with an efficiency of >80% and it is displayed with a binning of 50 ps. The evaluation of the fit function (orange) yields *g* ^(2)^(0) = 6.11(9)% including a correction for dark counts of the detectors. Similar values have been determined for other QDs on this sample and only minor bunching is found on time scales of up to ±2.5 μs.

In sum, the QDs show comparably promising results as their counterparts grown on a linearly graded MMB in terms of brightness, linewidth and shape, FSS, decay time and single-photon purity.

The second investigated structure is a circular Bragg grating (bullseye) cavity realized via a flip-chip process. Such a cavity design has been demonstrated with QDs emitting in the near-infrared spectral region [29, 30] and recently also for QDs emitting in the telecom O-band [31]. The approach has proven appealing for the realization of high brightness nonclassical light sources.

By reducing the inverse/capping layer thickness to 20 nm/180 nm and utilizing an AlGaAs sacrificial layer, a 360 nm thick membrane can be produced. This allows for sufficient confinement in the growth direction via total internal reflection. The complete layer structure is sketched in Figure 5E and the SEM image in Figure 5F shows a processed Bullseye structure after electron-beam lithography and chemical dry etching. *μ*-PL measurements were performed to investigate the cavity mode, ensuring its uniform feeding by high power above band-gap pumping. The spectral position of the fundamental mode for different trench widths is plotted in Figure 5G. For smaller values, a saturation behavior of the resonance wavelength is observed, while for increasing trench widths the cavity mode starts to blue-shift. This trend is verified by finite-difference time-domain simulations. However, a small offset between simulations and measurements is observed. This can possibly be explained by the simplified and averaged refractive index used in the simulations. In contrast, the real refractive index is highly dependent on the material composition and temperature. Furthermore, a small variation of the membrane or Al_2_O_3_ layer thickness can yield an additional small difference. An exemplary *μ*-PL spectrum of a cavity peak around 1550 nm (trench width of 240 nm) and a Q-factor of 160 (simulated Q-factor of 240) is displayed in Figure 5H, thus demonstrating the feasibility of fabricating Bullseye cavities in the telecom C-band based on the jump-convex-inverse MMB design. In the future, in order to achieve a perfect spatial overlap between a preselected QD and the fundamental cavity mode, a deterministic approach similar to Ref. [32] will be exploited as this can readily be transferred to the telecom wavelengths [33].

## 5 Conclusion and outlook

We realized the MOVPE growth of a thin-film InGaAs MMB with a thickness as small as 180 nm on a GaAs substrate, by optimizing a nonlinear grading profile designed for efficient plastic relaxation. Furthermore, we demonstrated that the strain on InAs QDs placed inside a respective InGaAs matrix can be adjusted to obtain telecom C-band photons. The MMB/QD structure was subsequently grown directly on a GaAs/AlAs DBR. This sample exhibits high surface quality and allowed us to demonstrate single-photon emission. Finally, the structure was integrated into a Bullseye cavity to prove its compatibility with highly appealing photonic resonator designs. This progress in MMB growth unlocks numerous approaches for the fabrication of high quality single-photon sources at 1550 nm.

## Supplementary Material

The online version of this article offers additional material: Experimental methods section, supplementary AFM-scans, description of further optimization steps, a mathematical expression of the convex-up grading function and exemplary reciprocal space maps. Furthermore, the minimum achievable MMB thickness and QD area density, as well as the spectroscopic study regarding the linewidth, fine-structure splitting and decay time of the QDs are presented.

## Supplementary Material

Supplementary Material Details

## References

[1] Michler P. (2017). *Quantum Dots for Quantum Information Technologies*.

[2] Liao S.-K., Yong H.-L., Liu C. (2017). Long-distance free-space quantum key distribution in daylight towards inter-satellite communication. Nat. Photonics.

[3] Agrawal G. P. (2012). *Fiber-Optic Communication Systems*.

[4] Takemoto K., Sakuma Y., Hirose S., Usuki T., Yokoyama N. (2004). Observation of exciton transition in 1.3–1.55 μm band from single InAs/InP quantum dots in mesa structure. Jpn. J. Appl. Phys..

[5] Skiba-Szymanska J., Stevenson R. M., Varnava C. (2017). Universal growth scheme for quantum dots with low fine-structure splitting at various emission wavelengths. Phys. Rev. Appl..

[6] Ledentsov N., Shchukin V., Kettler T. (2007). MBE-grown metamorphic lasers for applications at telecom wavelengths. J. Cryst. Growth.

[7] Semenova E., Hostein R., Patriarche G. (2008). Metamorphic approach to single quantum dot emission at 1.55 μm on GaAs substrate. J. Appl. Phys..

[8] Müller T., Skiba-Szymanska J., Krysa A. (2018). A quantum light-emitting diode for the standard telecom window around 1550 nm. Nat. Commun..

[9] Anderson M., Müller T., Skiba-Szymanska J. (2020). Gigahertz-clocked teleportation of time-bin qubits with a quantum dot in the telecommunication C band. Phys. Rev. Appl..

[10] Ajayan J., Nirmal D., Mohankumar P. (2019). GaAs metamorphic high electron mobility transistors for future deep space-biomedical-millitary and communication system applications: a review. Microelectron. J..

[11] Philipps S. P., Guter W., Welser E. (2012). Present status in the development of III–V multi-junction solar cells. *Next Generation of Photovoltaics*.

[12] Sorokin S., Klimko G., Sedova I. (2016). Peculiarities of strain relaxation in linearly graded In_x_Ga_1−x_As/GaAs (001) metamorphic buffer layers grown by molecular beam epitaxy. J. Cryst. Growth.

[13] Paul M., Olbrich F., Höschele J. (2017). Single-photon emission at 1.55 μm from MOVPE-grown InAs quantum dots on InGaAs/GaAs metamorphic buffers. Appl. Phys. Lett..

[14] Olbrich F., Höschele J., Müller M. (2017). Polarization-entangled photons from an InGaAs-based quantum dot emitting in the telecom C-band. Appl. Phys. Lett..

[15] Zeuner K. D., Jöns K. D., Schweickert L. (2021). On-demand generation of entangled photon pairs in the telecom C-band with InAs quantum dots. ACS Photonics.

[16] Nawrath C., Olbrich F., Paul M., Portalupi S., Jetter M., Michler P. (2019). Coherence and indistinguishability of highly pure single photons from non-resonantly and resonantly excited telecom C-band quantum dots. Appl. Phys. Lett..

[17] Nawrath C., Vural H., Fischer J. (2021). Resonance fluorescence of single In(Ga)As quantum dots emitting in the telecom C-band. Appl. Phys. Lett..

[18] Senellart P., Solomon G., White A. (2017). High-performance semiconductor quantum-dot single-photon sources. Nat. Nanotechnol..

[19] Matthews J., Mader S., Light T. (1970). Accommodation of misfit across the interface between crystals of semiconducting elements or compounds. J. Appl. Phys..

[20] Kujofsa T., Yu W., Cheruku S. (2012). Plastic flow and dislocation compensation in ZnS_y_ Se_1−y_/GaAs (001) heterostructures. J. Electron. Mater..

[21] Andrews A., Speck J., Romanov A., Bobeth M., Pompe W. (2002). Modeling cross-hatch surface morphology in growing mismatched layers. J. Appl. Phys..

[22] Rodriguez B., Millunchick J. (2004). The role of morphology in the relaxation of strain in InGaAs/GaAs. J. Cryst. Growth.

[23] Sasaki T., Suzuki H., Sai A. (2011). Growth temperature dependence of strain relaxation during InGaAs/GaAs (001) heteroepitaxy. J. Cryst. Growth.

[24] DenBaars S., Maa B., Dapkus P., Danner A., Lee H. C. (1986). Homogeneous and heterogeneous thermal decomposition rates of trimethylgallium and arsine and their relevance to the growth of GaAs by MOCVD. J. Cryst. Growth.

[25] Ceschin A. M., Massies J. (1991). Strain induced 2D–3D growth mode transition in molecular beam epitaxy of InxGa1t-xAs on GaAs (001). J. Cryst. Growth.

[26] Goldberg Yu A., Schmidt N., Levinshtein M. E., Rumyantsev S., Shur M. (1999). *Handbook Series on Semiconductor Parameters*.

[27] Xie Q., Madhukar A., Chen P., Kobayashi N. P. (1995). Vertically self-organized InAs quantum box islands on GaAs (100). Phys. Rev. Lett..

[28] Carmesin C., Olbrich F., Mehrtens T. (2018). Structural and optical properties of InAs/(In)GaAs/GaAs quantum dots with single-photon emission in the telecom c-band up to 77 k. Phys. Rev. B.

[29] Liu J., Su R., Wei Y. (2019). A solid-state source of strongly entangled photon pairs with high brightness and indistinguishability. Nat. Nanotechnol..

[30] Wang H., Hu H., Chung T.-H. (2019). On-demand semiconductor source of entangled photons which simultaneously has high fidelity, efficiency, and indistinguishability. Phys. Rev. Lett..

[31] Kolatschek S., Nawrath C., Bauer S. (2021). Bright Purcell enhanced single-photon source in the telecom O-band based on a quantum dot in a circular Bragg grating. Nano Lett..

[32] Kolatschek S., Hepp S., Sartison M., Jetter M., Michler P., Portalupi S. L. (2019). Deterministic fabrication of circular Bragg gratings coupled to single quantum emitters via the combination of in-situ optical lithography and electron-beam lithography. J. Appl. Phys..

[33] Sartison M., Engel L., Kolatschek S. (2018). Deterministic integration and optical characterization of telecom O-band quantum dots embedded into wet-chemically etched Gaussian-shaped microlenses. Appl. Phys. Lett..

