# Supplementary material for: Thin-film InGaAs metamorphic buffer for telecom C-band InAs quantum dots and optical resonators on GaAs platform
Source: Nanophotonics. 2022 Feb 8;11(6):1109–16. doi: 10.1515/nanoph-2021-0552 (PMC11501805; doi:10.1515/nanoph-2021-0552)
Supplement: Supplementary file 1 — Supplementary Material Details [file j_nanoph-2021-0552_suppl.pdf]

Supplementary material for “Thin-Film InGaAs Metamorphic Buffer for Telecom C-band InAs Quantum Dots and Optical Resonators on GaAs Platform”

Methods

The structures were grown in a commercial AIX-200 horizontal metal-organic vapor-phase epitaxy (MOVPE) system at a pressure of 100 mbar using the standard precursors trimethylgallium (TMGa), trimethylindium (TMIn), trimethylaluminum (TMAl) and arsine (AsH_3). We used exactly oriented (100) GaAs substrates and deposited a GaAs buffer to provide a high surface quality for the subsequent layers.

The compositional change within the $In_xGa_{1-x}As$ MMB in this work was realized by fixing the TMGa flux and adjusting the TMIn flux. This leads to a minor distortion of the grading profiles, because the deposition speed is slightly increased at higher indium contents (approx. 17 % increase between $In_{27.5}GaAs$ and $In_{38}GaAs$). However, this should have a negligible impact on the conclusions made for the comparison between different profiles. QDs were grown by depositing InAs for 4 s with a TMIn-flux of 9.6 µmol/min. Their growth temperature was 595 °C for the samples measured in Fig .4 and 550 °C for the DBR structure displayed in Fig. 5.

Atomic force microscopy (AFM) was used to investigate the surface topology and to determine its roughness given by the root mean square (RMS). This was our main criterion for the crystal quality of the MMB layers, because surface roughness prominently influences QD growth, excitation/extraction efficiency and the compatibility with nano-structuring techniques. All scans are depicted with the horizontal axis being aligned to the intersection of the surface with the Ga/In-facets ($[011]/[0\bar{1}\bar{1}]$) and the vertical axis being aligned to the respective As-facets ($[0\bar{1}1]/[01\bar{1}]$).

We recorded reciprocal space maps (RSM) in a high-resolution X-ray diffractometer (XRD) to determine the composition and relaxation inside MMB layers with a method similar to Ref. [1].

Deposition rates were calibrated by measuring layer thicknesses with a combination of scanning electron microscopy (SEM) and X-ray reflectivity (XRR).

In order to investigate the QD emission characteristics, photoluminescence (PL) or micro-photoluminescence (µ-PL) measurements were performed at 4 K, depending if an ensemble of QDs or a single QD is addressed, respectively. The dots were optically excited by a continuous wave (cw) laser above the bandgap of the surrounding matrix material. The QD emission is collected by the same objective used to focus the excitation laser and guided into a spectrometer. The ensemble PL measurements displayed in Fig. 4 are acquired using a 0.5 m spectrometer (Acton SP2500i equipped with a 150 mm^{-1} grating with a Andor iDus InGaAs 1.7 µm CCD) with a resolution of ca. 0.35 nm at 1550 nm. The µ-PL measurements displayed in Figs. 5C and D are acquired using a 0.5 m spectrometer (Acton SP2500i equipped with a 830 mm^{-1} grating with a OMA V InGaAs CCD from Princeton Instruments) with a resolution of ca. 0.08 nm at 1550 nm.

To measure the second-order auto-correlaction function $g^{(2)}(\tau)$, a single QDs transition is filtered with a spectral width of ≈5 GHz and coupled to a fiber-based Hanbury-Brown and Twiss type setup consisting of a beam splitter and two superconducting nanowire single-photon detectors (SNSPDs) by Single Quantum with a detection efficiency of >80 %. The full detection system including the subsequent time tagging electronics exhibit a temporal resolution of ≈45 ps. The measurement displayed in the main text has been performed with a mean count rate of 40 kcps and 36 kcps on the two detectors, an integration time of 45 min and is displayed with a time binning of 50 ps.

AFM scans complementing Fig. 2C

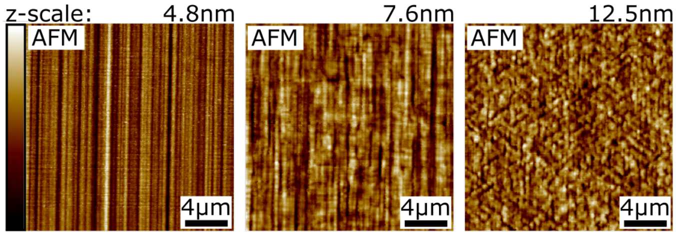

Fig. S1. Growth Stage I: AFM scans of InGaAs layers grown at a temperature of 580 °C. left, 6.0 µmol/min: cross-hatch with dominant $[0\bar{1}1]$ direction leads to streaky topography. center, 8.4 µmol/min: mostly balanced cross-hatch along [011] and $[0\bar{1}1]$ direction. right, 11.5 µmol/min: ordering along [001] and [010] directions.

Fig. S1 shows three AFM scans that are associated with Fig. 2C in the main text. They exemplify the progression of the surface structure for increasing TMIn flux. The typical $[011]/[0\bar{1}1]$ cross-hatch pattern is dominated by the $[0\bar{1}1]$ direction for lower fluxes (Fig. S1, left) and becomes more balanced at medium values (Fig. S1, center). However, a further increase of the TMIn flux causes an unusual patterning in [001] and [010] directions (Fig. S1, right).

Further optimization steps

In the main text only the most impactful optimization steps are outlined. Three additional, minor steps will be discussed in the following.

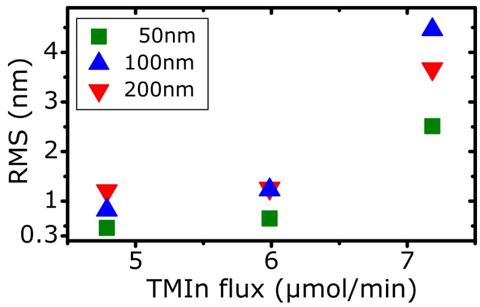

Fig. S2. Growth Stage I: Increase in surface roughness of InGaAs using a TMIn flux around 6.0 μmol/min and different thicknesses at a growth temperature of 710 °C.

In between the investigation of the surface RMS dependence on the TMIn flux in Fig. 2A and the temperature variation in Fig. 2B, we changed the layer thickness to 100 nm, because the roughening due to the transition between 2D and 3D growth is most pronounced there. The absolute difference in RMS between using 6.0 µmol and 7.2 µmol of TMIn flux is largest for 100 nm thick layers ($\Delta RMS_{100} = 3.21\,nm$), compared to 50 nm ($\Delta RMS_{50} = 1.86\,nm$) and 200 nm ($\Delta RMS_{200} = 2.41\,nm$) as displayed in Fig. S2.

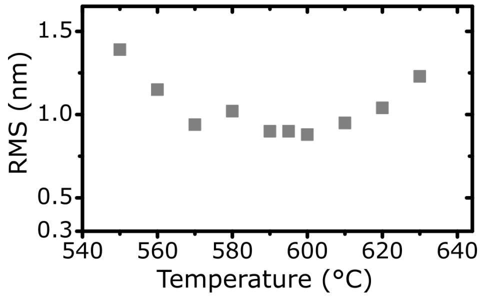

Fig. S3. Growth Stage I: Temperature dependent roughness of InGaAs with a TMIn flux of 8.4 µmol/min.

Fine-tuning of the growth temperature was performed after the determination of the final TMIn flux of 8.4 µmol/min (cf. Fig. 2C. in the main text). The small step investigation shown in Fig. S3 reinforced that a minimum of the roughness had been found and yielded 595 °C as the optimal setting for the temperature.

Simultaneously with the investigation of strain relaxation depending on layer thickness in Fig. 2D, we also analysed the surface roughening. The grey squares in Fig. S4 represent the previous relaxation data from Fig. 2D, showing saturation for higher thicknesses as described in the main text. In contrast, the respective RMS (blue upwards triangles) keeps increasing approximately linearly and shows no saturation. We have observed this linear dependence for InGaAs structures with a

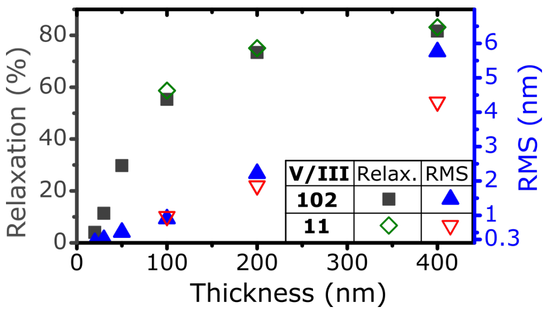

Fig. S4. Growth Stage I: Strain relaxation and surface roughness of $In_{27.4}GaAs$ with a V/III-ratio during growth of 100 and 11 at 595 °C for different thicknesses.

total thickness of up to 1.5 µm (not shown here). This makes it unlikely that the roughening is solely caused by the strain field of buried misfit dislocations [2]. A possible explanation could be that the combination of high indium content and low growth temperature leads to phase separation [3], which then stabilises the imbalance in growth speed between Ga-rich valleys and In-rich hillocks. Phase separation can be suppressed through enhanced surface mobility and intermixing at higher temperatures [4]. As the growth temperature is fixed at 595 °C here, we increased the surface mobility by reducing the V/III-ratio. We observed an improvement of the RMS, which is mainly visible for thicker layers (>50 nm), for a V/III-ratio of 11 without affecting the relaxation process (cf. Fig. S4, empty green diamonds and red downwards triangles). We adopted this reduced V/III-ratio for the further growth, because the final structure we are aiming for has a total thickness of 440 nm.

Convex-up grading function

We used the function of a quarter circle in the second quadrant for the convex-up grading section with the starting point being shifted appropriately. This means the TMIn flux as a function of growth time is given by

$$y(t) = y_0 + G \cdot \sqrt{1-(t-1)^2},$$

with y_0 being the flux of the jump step, G being the maximum grading and t going from 0 to 1.

Exemplary reciprocal space maps

Fig. S5 shows reciprocal space maps (RSMs) around the (400) and (422) reflex of a DBR sample. (cf. Figs 5A-D) measured via HRXRD. They show a slight offset in q_z direction, which is corrected in the analysis by calibrating relative to the GaAs peak (see Ref. [1]). Furthermore, the [400] map on the left is mostly used for tilt correction.

However, the lattice constant and strain situation inside the sample can be conveniently discussed on the basis of the RSM around the (422) reflexes shown on the right. It exhibits four distinct peaks, of which the two high-intensity ones at large q_x and q_z can be attributed to the GaAs substrate and the pseudomorphic AlAs inside the DBR. Thus, the remaining signal originates from the InGaAs layers inside the MMB. A

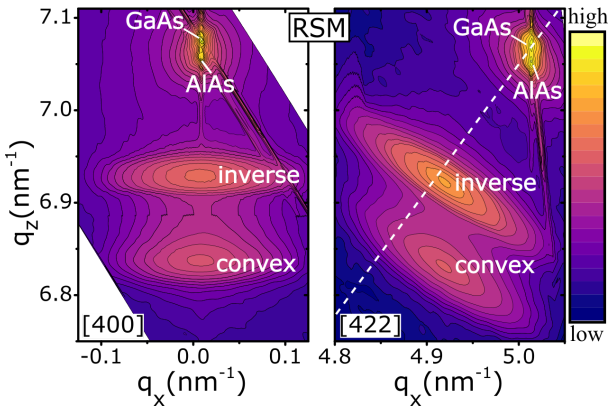

Fig. S5. Reciprocal space maps around the (400) (left) and (422) (right) reflex measured via HRXRD. The intensity peaks from the MMB are labelled according to their respective optimization step (3) and (4). The dashed line in the right panel marks the coordinates of full relaxation.

positioning at low q_x and q_z signifies a high indium content for one of the peaks. Additionally, it is furthest away from the dashed line in [422] direction, which marks full relaxation at varying lattice constants. Therefore, we can identify it as the residually strained (70 %) relaxation) top part of the convex layer and it is labelled accordingly. Consequently, the last peak represents a mostly relaxed (96 %) crystal structure with a medium indium content, which fits to the inverse layer. Both peaks possess the same q_x coordinate and thus their in-plane lattice constant matches as intended in our design. Furthermore, we observe a significant broadening in diagonal direction. This indicates a pronounced mosaicity inside the InGaAs structure. Notably missing is the signal from the low indium area between the GaAs position and inverse-layer-peak, which is omitted because of the jump in content for the GaAs/InGaAs transition (cf. Fig. 1).

Minimal Achievable Thickness

The total MMB thicknesses shown in the main text were set by the necessary thickness of the intended structures i.e. a DBR structure with λ-cavity thickness and a membrane for the realization of a circular Bragg grating cavity. However, the achievable minimal thickness of the presented jump-convex-inverse design is an interesting characteristic. Here, the reduction potential of the jump, convex and inverse step have to be evaluated separately and are discussed in the following. The thickness of the inverse layer can be readily modified without disturbing the strain engineering, as it has been shown for the bullseye structure (60 nm $\rightarrow$ 20 nm). However, some adaptation of the QD deposition might be necessary in order to achieve best results, since the optimization of the growth parameters has been performed for the thicker MMB. We have observed a slight decrease of the optical quality for QDs grown on the thinner inverse layer and deposition directly on the convex layer has shown to have a significant detrimental effect. The exact limit of the later effect ($>$ 0 nm, $<$ 20 nm) has not been investigated, but will form part of future works.
Reducing the thickness of the jump layer from 30 nm to 20 nm increases the roughness moderately by 10 %. Further reduction to 10 nm undermines the strain engineering and leads to 375 % roughening.
Notably, any modification of the jump+convex layer thickness changes the relaxation state (cf. Fig. 2D) and thus has to be compensated by the maximum indium composition of the grading to maintain the same effective lattice constant for the QD growth. The baseline is the lattice constant of $In_{0.285}GaAs$ given by a reference sample (30 nm jump, 130 nm inverse). The compensation procedure has been tested for a 100 nm thick convex layer. After increasing the final TMIn flux by 10 % we were only able to reach the lattice constant of $In_{0.250}GaAs$, while experiencing an RMS increase of approximately 10 %. Further investigation could be sensible in case a specific photonic structure necessitates a substantial MMB thickness reduction.

QD Area Density

In Fig. 5C of the main text we show a μ-PL map displaying a low QD area density. This result can be discussed in the context of other results in literature and applications necessitating a higher density.
In Ref. [5] an area density of optically active QDs of 1 x 10^7 cm^{-2} has been reported for a linear MMB structure. We have used similar QD growth parameters and obtained a comparable value of 3 x 10^6 cm^{-2}. In general, as shown for the standard GaAs substrate, the area density of InAs QDs can be easily controlled over a wide range by setting the V/III-ratio [6]. However, this technique might be complicated by the rougher MMB surface providing distinct nucleation sites. We have shown that a conveniently low area density to isolate individual emitters for single-photon applications can be established. Given the focus for these applications, we omitted an investigation or optimization to reach values in the $>$ 10^{10} cm^{-2} range necessary for laser applications. In this case one would certainly have to account for the broad ensemble spectrum of the QDs as depicted in Fig. 4.

Linewidth Evaluation via First-Order Coherence Function

As discussed in the main text, the linewidth of the QD transition peaks via a Gaussian fit to the spectrum is partially limited by the spectrometer resolution (see the methods section above for details on the measurement setup). Therefore, the linewidth evaluations are complemented by first-order coherence measurements using a free-space Michelson interferometer with a motorized delay stage (travel range $\pm$75 mm). The measurements are acquired under non-resonant, cw excitation under saturation conditions. To obtain the visibility, the count rate as a function of the relative path delay is fit with a sine function around a given delay position, and corrected for the detector dark counts and the non-unity visibility of the setup due to alignment imperfections ($\sim$ 3 %). The resulting trace of the visibility over the relative temporal delay τ is proportional to the first-order coherence function $g^{(1)}(\tau)$ and fit with the Fourier transform of a Voigt function [7] to account for both

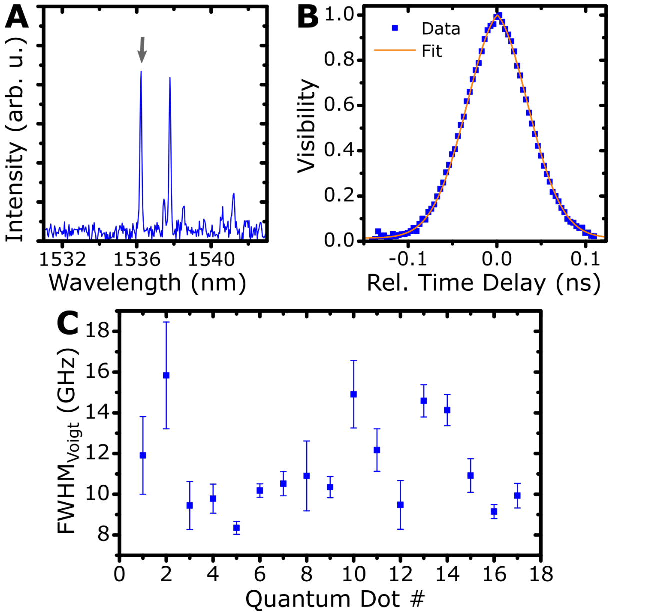

Fig. S6. Measurement of the first-order coherence function $g^{(1)}(\tau)$ under cw, non-resonant excitation in saturation. A: μ-PL spectrum of an exemplary QD transition. B: $g^{(1)}(\tau)$ measurement data (blue) and fit function (orange) on the transition marked with an arrow in panel A. C: Linewidth (FWHM) of 17 exemplary QD transitions calculated from the fit functions to the corresponding measurements.

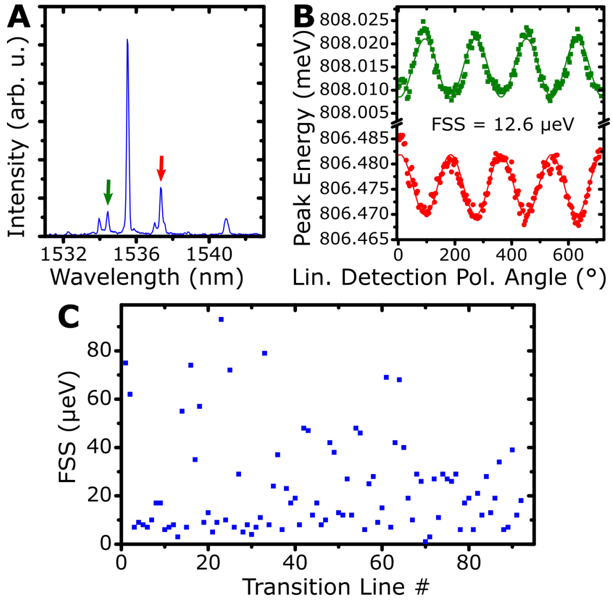

Fig. S7. Measurement of the FSS under cw, non-resonant excitation in saturation. A: Spectrum of an exemplary QD. B: Energetic position of the transition lines marked with the correspondingly colored arrows in panel A for varying linear detection polarization. C: FSS of the transition lines of 26 QDs.

homogeneous (Lorentzian) and inhomogeneous (Gaussian) broadening. The data (blue) and the fit function (orange line) of such a measurement are displayed for an exemplary QD in Fig. S6B with the corresponding $\mu-$PL spectrum in Fig. S6A. As it is expected for non-resonant pumping under saturation conditions, the transitions are dominated by inhomogeneous broadening. The ratio of homogeneous and inhomogeneous broadening and the absolute values compare well with QDs on linearly-graded MMBs [8]. The FWHM of the full Voigt profile calculated from the fit function is shown for 17 exemplary QDs in Fig. S6C with the error bars indicating the uncertainty calculated via error propagation from the 1σ confidence bound of the fit routine. The mean value results in 11.3 GHz (46.7 μeV) with a standard deviation of 2.2 GHz (9.1 μeV).

Evaluation of the Fine-Structure Splitting

The measurement procedure to evaluate the fine-structure splitting (FSS) is outlined in Fig. S7 for an exemplary QD. The spectrum, as displayed in Fig. S7A, under non-resonant, cw excitation at saturation, is acquired for 180 equally spaced linear detection polarization angles over a range of 720 °. The transition lines are individually fit with a Gaussian function and the resulting energetic peak position is plotted over the detection angle (see Fig. S7B). Two transition lines exhibiting oscillations with the same amplitude and a phase difference of 90 ° are a hallmark for a biexciton-exciton pair, as displayed in Fig. S7B. The FSS is twice the amplitude of the sinusoidal oscillation. Note that among the investigated 26 QDs, only 7 exhibited this behaviour. Therefore, also individual transition lines exhibiting an oscillation are included in the statistic shown in Fig. S7C yielding a mean value of 23.4 μeV and a standard deviation of 20.6 μeV. Thus the low average FSS values of GaAs-based QDs emitting in the telecom C-band based on a linearly-graded MMB [5], [9] are found for their counterparts on a non-linearly graded MMB as well. Note that transition lines with no resolvable oscillations are excluded from this evaluation.

Evaluation of the Decay Time

The $\mu-$PL spectrum displayed in Fig. S8 shows an exemplary QD under pulsed, resonant excitation. While the spectrum is taken under saturation conditions, the decay trace (Fig. S8B) is measured under weak excitation via time-correlated single-photon counting (TCSPC). After a sharp rise of the detected counts relative to the trigger pulse of the excitation laser, a small plateau is visible due to the relaxation time jitter of the charge carriers into the QD potential leading to the formation of the excitonic state in question. This is followed by an exponential decay. The orange, solid line represents the fit function which is found to be mono-exponential in the vast majority of the 13 exemplary QDs investigated. The corresponding $1/e$-decay times are displayed in Fig. S8C. The mean value is found to be 1.39 ns with a standard deviation of 0.16 ns and compares well to the QDs based on a linearly-graded MMB [5], [8]. Note that equating the decay time with the radiative lifetime T_1 implies the assumption of negligible non-radiative decay channels.

References

[1] Chauveau, J.-M., Androussi, Y., Lefebvre, A., Di Persio, J. & Cordier, Y. Indium content measurements in metamorphic high electron mobility

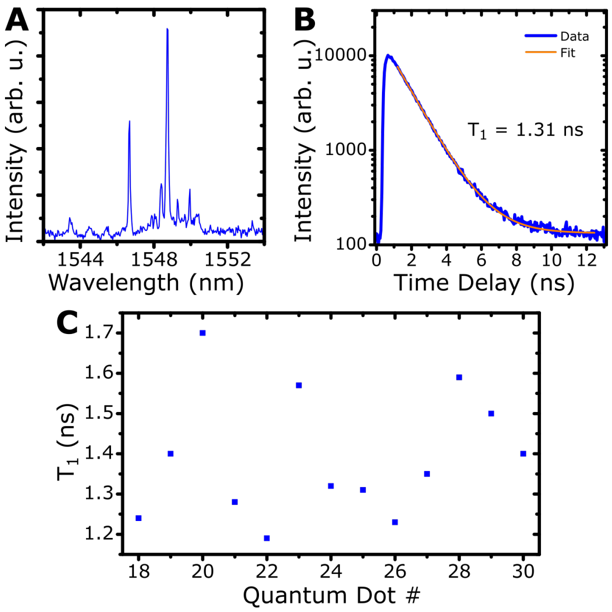

Fig. S8. Decay time measurements under pulsed, non-resonant excitation. A: $\mu-$PL spectrum of an exemplary QD in saturation. B: TCSPC data measurement data (blue) and fit (orange) of the decay time of the brightest transition line in panel A under weak excitation. C: Decay times of 13 exemplary QDs.

transistor structures by combination of x-ray reciprocal space mapping and transmission electron microscopy. *Journal of Applied Physics* **93**, 4219–4225 (2003).

[2] Zhou, X. *et al.* Strain-field induced crosshatch formation during molecular beam epitaxy of InGaAs/GaAs films. *MRS Online Proceedings Library Archive* **312** (1993).

[3] Lee, K. E. & Fitzgerald, E. A. High-quality metamorphic compositionally graded InGaAs buffers. *Journal of Crystal Growth* **312**, 250–257 (2010).

[4] Quitoriano, N. J. & Fitzgerald, E. A. Relaxed, high-quality InP on GaAs by using InGaAs and InGaP graded buffers to avoid phase separation. *Journal of Applied Physics* **102**, 033511 (2007).

[5] Paul, M. *et al.* Single-photon emission at 1.55 μ m from MOVPE-grown InAs quantum dots on InGaAs/GaAs metamorphic buffers. *Applied Physics Letters* **111**, 033102 (2017).

[6] Li, S. *et al.* InAs/GaAs quantum dots with wide-range tunable densities by simply varying V/III ratio using metal-organic chemical vapor deposition. *Nanoscale research letters* **8**, 1–5 (2013).

[7] Versteegh, M. A. M. *et al.* Observation of strongly entangled photon pairs from a nanowire quantum dot. *Nature Communications* **5**, 5298 (2014). URL `http://www.nature.com/articles/ncomms6298`.

[8] Nawrath, C. *et al.* Coherence and indistinguishability of highly pure single photons from non-resonantly and resonantly excited telecom C-band quantum dots. *Applied Physics Letters* **115**, 023103 (2019).

[9] Olbrich, F. *et al.* Polarization-entangled photons from an InGaAs-based quantum dot emitting in the telecom C-band. *Applied Physics Letters* **111**, 133106 (2017).
